# Interferon Tau Affects Mouse Intestinal Microbiota and Expression of IL-17

**DOI:** 10.1155/2016/2839232

**Published:** 2016-08-17

**Authors:** Wenkai Ren, Shuai Chen, Liwen Zhang, Gang Liu, Tarique Hussain, Xiao Hao, Jie Yin, Jielin Duan, Bie Tan, Guoyao Wu, Fuller W. Bazer, Yulong Yin

**Affiliations:** ^1^Key Laboratory of Agro-Ecological Processes in Subtropical Region, Institute of Subtropical Agriculture, Chinese Academy of Sciences, Observation and Experiment Station of Animal Nutrition and Feed Science in South-Central China, Ministry of Agriculture, Hunan Provincial Engineering Research Center for Healthy Livestock and Poultry Production, Changsha, Hunan 410125, China; ^2^University of the Chinese Academy of Sciences, Beijing 10008, China; ^3^Jinan First People's Hospital, Shandong 250011, China; ^4^Department of Animal Science, Texas A&M University, 2471 TAMU, College Station, TX 77843-2471, USA

## Abstract

This study was conducted to explore the effects of interferon tau (IFNT) on the intestinal microbiota and expression of interleukin 17 (IL-17) in the intestine of mice. IFNT supplementation increased microbial diversity in the jejunum and ileum but decreased microbial diversity in the feces. IFNT supplementation influenced the composition of the intestinal microbiota as follows: (1) decreasing the percentage of* Firmicutes* and increasing* Bacteroidetes* in the jejunum and ileum; (2) enhancing the percentage of* Firmicutes* but decreasing* Bacteroidetes* in the colon and feces; (3) decreasing* Lactobacillus* in the jejunum and ileum; (4) increasing the percentage of* Blautia*,* Bacteroides*,* Alloprevotella,* and* Lactobacillus* in the colon; and (5) increasing the percentage of* Lactobacillus*,* Bacteroides*, and* Allobaculum*, while decreasing* Blautia* in the feces. Also, IFNT supplementation decreased the expression of IL-17 in the intestines of normal mice and of an intestinal pathogen infected mice. In conclusion, IFNT supplementation modulates the intestinal microbiota and intestinal IL-17 expression, indicating the applicability of IFNT to treat the intestinal diseases involving IL-17 expression and microbiota.

## 1. Introduction

Interferon tau (IFNT) is produced by trophectoderm cells of conceptuses of ruminant species and is the maternal recognition of the pregnancy signal. Besides its critical roles in implantation and establishment of pregnancy in ruminants [1, 2], it has a plethora of physiological functions in various cell types such as macrophages, lymphocytes, and epithelial cells in humans and mice [3–5]. It is a type I interferon (IFN), which includes IFN alpha (IFNA), IFN beta (IFNB), IFN delta (IFND), and IFN omega (IFNW). After binding to a common receptor, IFNA receptor 1 (IFNAR1), and IFNAR2, type I IFNs affect the production of inflammatory cytokines such as interleukin- (IL-) 1*β* and tumor necrosis factor *α* (TNF-*α*) [6, 7]. Thus, type I IFNs have widely recognized roles in inflammatory diseases, such as experimental allergic encephalomyelitis, multiple sclerosis, and spontaneous autoimmune diabetes [5, 8–10]. Notably, unlike other members of type I IFN family, IFNT has few adverse effects and low cytotoxicity even at high dosages [11, 12], suggesting its therapeutic potential as an alternative to other type I IFNs due to its anti-inflammatory effects. Recent compelling findings about the anti-inflammatory effects of IFNT include lower NLRP3 (nucleotide-binding oligomerization domain-like receptor, pyrin domain-containing 3) inflammasome-driven IL-1*β* secretion by human macrophages [4], mitigation of obesity-associated systemic tissue inflammation in mice [5], and promotion of Th2 biased immune response in mice [9].

The influence of IFNT on intestinal microbiota is unknown. The intestinal microbiota provides important benefits for the development of immune responses; however, the disturbances in the intestinal microbiota are associated with numerous chronic inflammatory diseases [13, 14]. Also, the effect of IFNT on expression of IL-17 in the intestine is not known. The potential effect of IFNT on expression of IL-17 is important as IL-17 promotes local chemokine production to recruit monocytes and neutrophils to sites of inflammation that leads to development and pathogenesis of various autoimmune diseases, including rheumatoid arthritis, psoriasis vulgaris, multiple sclerosis, and inflammatory bowel diseases [15, 16]. In this study, the intestinal microbiota and expression of IL-17 in the intestine were explored after two weeks of IFNT supplementation in a mouse model. The hypothesis is that IFNT supplementation alters intestinal microbiota and intestinal innate immunity in mouse model.

## 2. Materials and Methods

### 2.1. Bacterial Strains

This study used the* Escherichia coli* F4-producing strain W25K (hereafter referred as ETEC; O149:K91, K88ac; LT, STb, EAST), which was originally isolated from a piglet with diarrhea [17].

### 2.2. IFNT Supplementation for Mice

This study was conducted according to the guidelines of the Laboratory Animal Ethical Commission of the Chinese Academy of Sciences. ICR (Institute for Cancer Research) mice (six weeks of age) were purchased from SLAC Laboratory Animal Central (Changsha, China). The mice were housed individually in a pathogen-free animal vivarium (temperature, 25°C; relative humidity, 53%; 12 h dark/12 h light) and had free access to a standard rodent diet [18] and drinking water. After three days of accommodation, mice were assigned randomly into two groups (IFNT and control; *n* = 10/group). Mice in the control group were fed the basal diet [18] and normal water, while mice in IFNT group were fed the basal diet and water containing recombinant IFNT (40 *μ*g/L) for two weeks. The effective supplemental dosage of IFNT was established in previous study [5, 19]. At the end of the two weeks of experimental period, mice were sacrificed to collect contents of the lumens of the jejunum, ileum, and colon, as well as feces. The tissues including jejunum, ileum, and colon were also collected. Feed and water intake and body weight gain were monitored throughout the experiment. Samples were collected and stored at −80°C until processed.

### 2.3. ETEC Infection of Mice

After three days of accommodation to the conditions of the vivarium, ICR mice were assigned randomly into two groups (ETEC and IFNT+ETEC; *n* = 10/group). Mice in IFNT+ETEC group were fed the basal diet and recombinant IFNT-supplemented water (40 *μ*g/L) for two weeks, while mice in ETEC group were fed the basal diet and had normal water. After two weeks of feeding, mice in both groups were inoculated with 10^8^ CFUs of ETEC W25K by oral gavage. At 6 hours after infection, all active mice were sacrificed to collect the jejunum, and the samples were stored at −80°C until processed.

### 2.4. 16S rDNA Sequencing with Illumina MiSeq Sequencing

DNA was extracted from the luminal contents of the jejunum, ileum and colon, and feces using the Qiagen QIAamp DNA Stool Mini Kit according to the protocol for isolation of DNA. Equal amounts of DNA from six different mice were pooled to generate one common sample for each type of sample (i.e., control versus IFNT, intestinal source, and feces). The V4-V5 region of the bacterial 16S ribosomal RNA gene was amplified by PCR using primers 515F 5′-barcode-GTGCCAGCMGCCGCGG-3′ and 907R 5′-CCGTCAATTCMTTTRAGTTT-3′, where barcode is an eight-base sequence unique to each sample. Illumina MiSeq sequencing and general data analyses were performed by a commercial company (Biotree, Shanghai, China). Miseq PE Libraries, Miseq Sequencing, and further analyses were based on previous work [20].

### 2.5. RT-PCR

Total RNA was isolated from liquid nitrogen frozen and ground jejunum, ileum, and colon using TRIZOL regent (Invitrogen, USA) and then treated with DNase I (Invitrogen, USA) according to the manufacturer's instructions. Synthesis of the first strand (cDNA) was performed using oligo (dT) 20 and Superscript II reverse transcriptase (Invitrogen, USA). Primers were selected according to previous references [18, 21]. *β*-actin was used as an internal control to normalize expression of target gene transcripts. The RT-PCR experiment was conducted according to previous studies [18, 21].

### 2.6. Statistical Analyses

Data shown are the means ± the standard error of the mean (SEM). All statistical analyses for data were performed using SPSS 16.0 software (Chicago, IL, USA). Data were analyzed for the two treatment groups using Student's* t*-test. Differences of *P* < 0.05 are considered significant.

## 3. Results 

### 3.1. IFNT Treatment Increases Feed Intake

**Figure 1:**
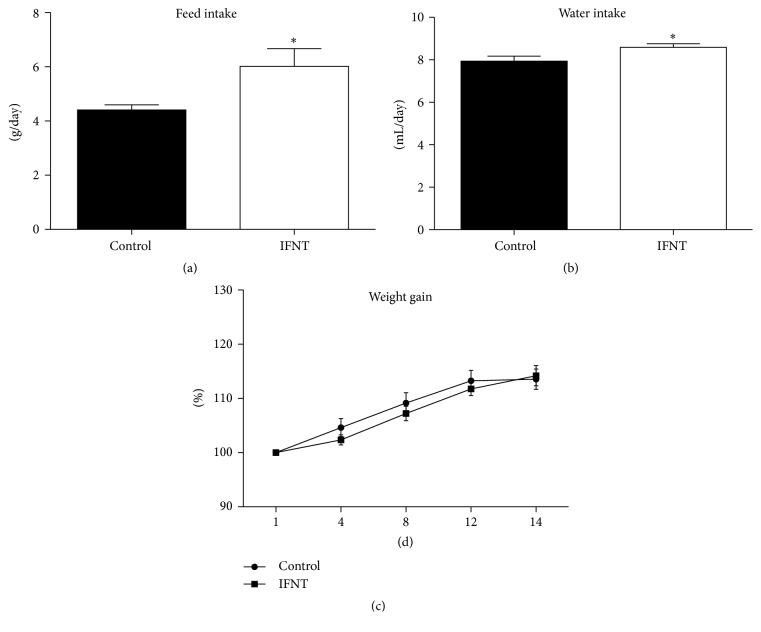
IFNT supplementation has little effect on mouse body weight. (a) Average feed intake in the control and IFNT-supplemented mice (*n* = 10). (b) Average water intake for control and IFNT-supplemented mice (*n* = 10). (c) Relative body weight gains for control and IFNT-supplemented mice (*n* = 10). Control mice were fed the basal diet and normal water, while mice in IFNT group were fed the basal diet and IFNT-supplemented water for two weeks. The asterisk (*∗*) indicates a statistically significant difference between two treatment groups (*P* < 0.05). Data were analyzed using Student's* t*-test.

To investigate the effect of IFNT supplementation on mouse growth performance, feed intake, water intake, and body weight were monitored in IFNT-supplemented mice and control mice. With two weeks of IFNT supplementation, the averages for feed intake and water intake for IFNT-supplemented mice were significant (*P* < 0.05) higher than for control mice (Figures 1(a) and 1(b)). However, IFNT supplementation had no significant effect on body weight of mice (Figure 1(c)).

### 3.2. Changes in Bacterial Diversity of the Intestinal Microbiota Associated with IFNT Supplementation

To explore the influence of IFNT supplementation on the intestinal microbiota, we analyzed the intestinal microbiota at end of two weeks of IFNT supplementation with 16S rDNA sequencing (Table 1). For microbiota in the jejunum, both Shannon and Simpson indices demonstrated that the diversity of microbiota in mice with IFNT supplementation was higher than the control mice, while the richness indices (Ace and Chao) suggested that the community richness in IFNT-supplemented and control mice was similar (Table 1). For the microbiota in the ileum, the diversity of microbiota (Shannon and Simpson) and richness indices (Ace) for mice with IFNT supplementation were higher than that of control mice (Table 1). For the microbiota in the colon, the diversity of microbiota (Shannon) and richness indices (Ace and Chao) were similar for IFNT-supplemented and control mice (Table 1). For the fecal microbiota, microbial diversity (Shannon and Simpson) in mice with IFNT supplementation was lower than for control mice, while the community richness (Ace and Chao) for IFNT-supplemented mice was similar to that for control mice (Table 1). Collectively, IFNT supplementation increases the diversity of microbiota in small intestine, while decreasing the diversity of microbiota in the feces.

### 3.3. IFNT-Associated Alterations in Intestinal Microbiota

The taxonomy of the intestinal microbiota was assessed using a taxon-dependent analysis and the RDP classifier. Seven phyla, including one candidate division (TM7), were found in the microbiota of the jejunum for all samples, including six phyla in the control mice and seven phyla in mice with IFNT supplementation. Eight phyla were found in the microbiota of the ileum of all samples, including six phyla in control mice and seven phyla in mice with IFNT supplementation. Ten phyla were found in the microbiota of the colon of all samples, including ten phyla in control mice and eight phyla in mice with IFNT supplementation. Ten phyla were found in the microbiota of the feces of all samples, including nine phyla in control mice and ten phyla in mice with IFNT supplementation.

**Figure 2:**
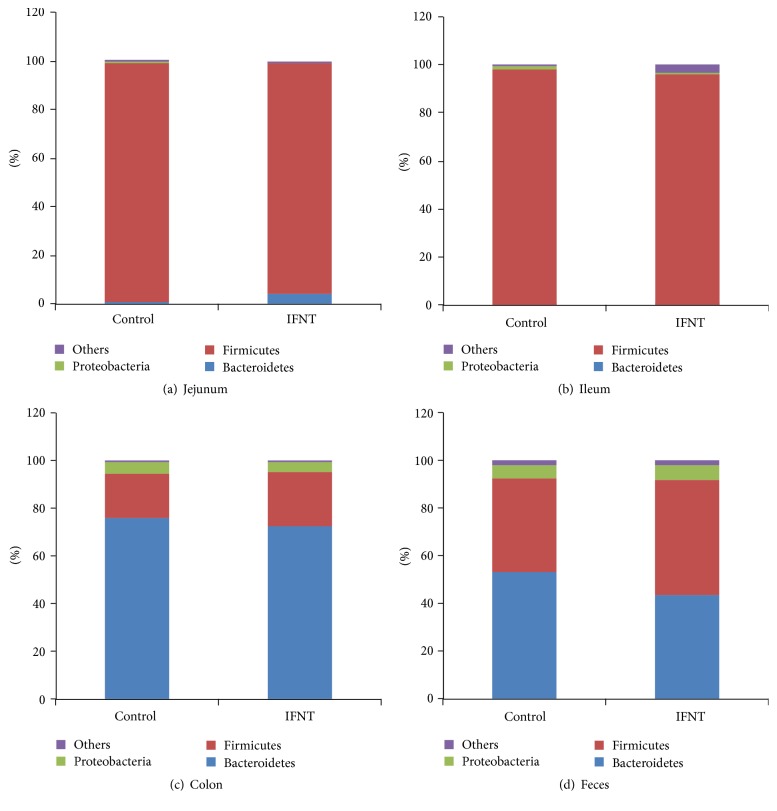
Composition of the intestinal microbiota at the phylum level after IFNT supplementation. (a) The microbial composition in the jejunum. (b) The microbial composition in the ileum. (c) The microbial composition in the colon. (d) The microbial composition in the feces.

For the jejunum, the two most abundant phyla in IFNT-supplemented mice, accounting for approximately 99% of all assigned sequence readings, were* Firmicutes* (94.5%) and* Bacteroidetes* (4.4%) (Figure 2(a)). In control mice, most abundant phyla were* Firmicutes* (97.6%) and* Bacteroidetes* (1.2%) (Figure 2(a)). For the ileum, the three most abundant phyla in IFNT-supplemented mice were* Firmicutes* (95.3%),* Candidate_division_TM7* (2.3%), and* Proteobacteria* (1.1%) (Figure 2(b)), while in control mice, they were* Firmicutes* (97.7%),* Proteobacteria* (1.5%), and* Bacteroidetes* (0.5%) (Figure 2(b)). For the microbiota in the colon, the three most abundant phyla in IFNT-supplemented mice were* Bacteroidetes* (72.2%),* Firmicutes* (23.0%), and* Proteobacteria* (4.1%) (Figure 2(c)), while they were* Bacteroidetes* (75.8%),* Firmicutes* (18.8%), and* Proteobacteria* (4.3%) in control mice (Figure 2(c)). For feces, the three most abundant phyla in IFNT-supplemented mice were* Bacteroidetes* (43.6%),* Firmicutes* (48.1%), and* Proteobacteria* (5.9%) (Figure 2(d)), while* Bacteroidetes* (53.2%),* Firmicutes* (39.2%), and* Proteobacteria* (5.3%) were most abundant for control mice (Figure 2(d)).

**Figure 3:**
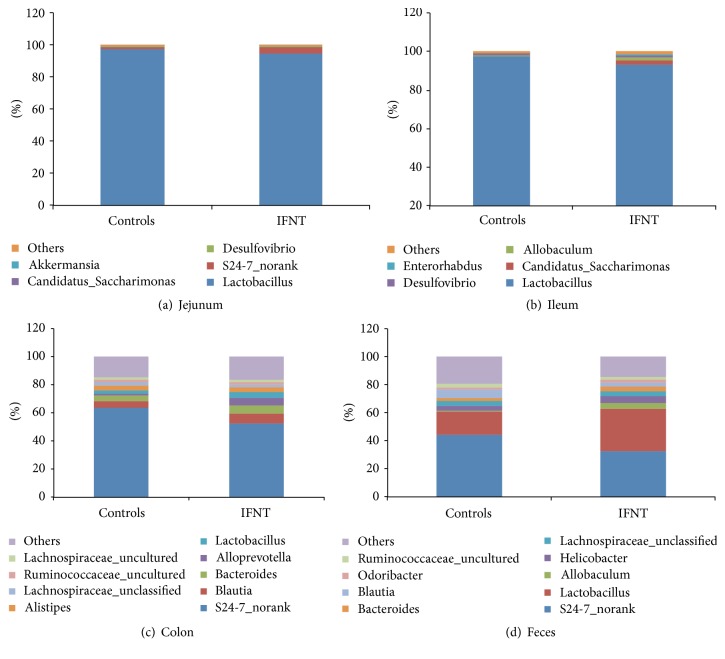
The composition of the intestinal microbiota at the genus level after IFNT supplementation. (a) The microbial composition in the jejunum. (b) The microbial composition in the ileum. (c) The microbial composition in the colon. (d) The microbial composition in the feces.

For the microbiota of the jejunum, the two most abundant genera in IFNT-supplemented mice, accounting for approximately 99% of all assigned sequence readings, were* Lactobacillus* (94.3%) and* S24-7_norank* (4.9%) (Figure 3(a)). In control mice, they were* Lactobacillus* (97.3%) and* S24-7_norank* (1.2%) (Figure 3(a)). For the ileum, the five most abundant genera in IFNT-supplemented mice were* Lactobacillus* (93.3%),* Candidatus-Saccharimonas* (2.3%),* Allobaculum* (1.2%),* Desulfovibrio* (1.1%), and* Enterorhabdus* (0.6%), while they were* Lactobacillus* (97.3%),* Candidatus-Saccharimonas* (0.3%),* Allobaculum* (0.1%),* Desulfovibrio* (1.5%), and* Enterorhabdus* (0.07%) in control mice (Figure 3(b)). For the microbiota in the colon, IFNT supplementation increased the percentages of* Blautia* (7.0% versus 5.1%),* Bacteroides* (6.4% versus 3.7%),* Alloprevotella* (5.2% versus 1.3%), and* Lactobacillus* (4.0% versus 2.5%), compared with control mice (Figure 3(c)). For the fecal microbiota, IFNT supplementation increased the percentages of* Lactobacillus* (30.0% versus 16.7%),* Bacteroides* (3.3% versus 1.8%), and* Allobaculum* (4.5% versus 0.4%), while decreasing the* Blautia* (2.7% versus 6.5%) compared with control mice (Figure 3(d)).

Collectively, IFNT supplementation affects the composition of intestinal microbiota in mice, especially those for the colon and feces.

### 3.4. IFNT Inhibits Expression IL-17 in the Intestine

**Figure 4:**
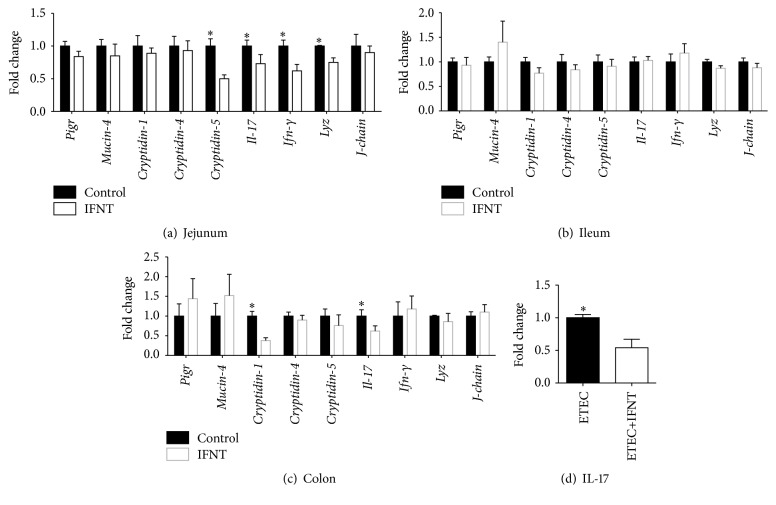
IFNT supplementation decreases expression of IL-17. (a) Expression of innate immune factors (*Pigr*,* Mucin-4*,* Cryptidin-1*,* Cryptidin-4*,* Cryptidin-5*,* Il-17*,* Ifn-γ*,* Lyz,* and* J-chain*) in the jejunum of mice (*n* = 10). (b) Expression of innate immune factors in the ileum of mice (*n* = 10). (c) Expression of innate immune factors in the colon of mice (*n* = 10). (d) IFNT decreases expression of IL-17 in the jejunum of mice following ETEC infection (*n* = 10). Data were analyzed using Student's* t*-test. An asterisk (*∗*) indicates a statistically significant difference between treatment groups (*P* < 0.05).

The effect of IFNT supplementation on activation of intestinal innate immunity in mice was further explored, focusing on the expression of polymeric immunoglobulin receptor (*Pigr*),* Mucin-4*,* Cryptidin-1*,* Cryptidin-4*,* Cryptidin-5*,* Il-17*, interferon gamma (*Ifn-γ*), lysozyme (*Lyz*), and* J-chain* in the jejunum, ileum, and colon [18, 21]. In the jejunum, IFNT supplementation significantly decreased the expression of* Cryptidin-5*,* Il-17*,* Ifn-γ*, and* Lyz*, while it had little effect on the expression of the other transcripts (Figure 4(a)). IFNT supplementation had no significant effect on the expression of those transcripts in the ileum of mice (Figure 4(b)). In the colon, IFNT supplementation significantly lowered the expression of* Cryptidin-1* and* Il-17* but had little effect on the expression of the other transcripts (Figure 4(c)). As IFNT supplementation decreased the expression of* Il-17* in the jejunum and colon, we further validated the effect of IFNT to decrease expression of IL-17 in ETEC infected mouse model. We found that ETEC infection promotes the* Il-17* expression in the mouse jejunum at 6 hours after infection (W. Ren and Y. Yin, unpublished results). After two weeks of IFNT supplementation, expression of* Il-17* in the jejunum was significantly lower in IFNT-supplemented mice, compared to that of nonsupplemented mice during ETEC infection (Figure 4(d)). Thus, IFNT supplementation reduces the expression of the inflammatory cytokine, IL-17, in the intestine of mice.

## 4. Discussion 

In this study, although two weeks of IFNT supplementation increases the mouse feed and water intake but has little effect on body weight of mice. Results of a previous study revealed that IFNT supplementation (8 *μ*g/kg BW/day) reduces body weight beginning at 3 weeks after IFNT supplementation in Zucker Diabetic Fatty rats, while lower dose of IFNT supplementation (4 *μ*g/kg BW/day) has no significant effect on body weight during 8 weeks of IFNT treatment [19], indicating that the effect of IFNT on body weight depends on dosage and duration of IFNT treatment. However, in a mouse model with high-fat or low-fat diet, 12 weeks of IFNT treatment does not significantly affect body weight [5]. However, IFNT supplementation has little effect on feed intake and water intake in those investigations [4, 5].

In the present study, IFNT supplementation increases the microbial diversity in the jejunum and ileum, while decreasing the microbial diversity in the feces of mice. The gut microbiota affects numerous biological functions [22, 23] and is linked to the pathogenesis of various diseases, such as obesity [24], cancer [25], and liver cirrhosis [26]. The influence of the gut microbiome on host physiological functions and the pathogenesis of disease in hosts may result from the activities of the microbiome and its metabolic products [22]. It is widely accepted that body weight is associated with the composition of intestinal microbiome and its metabolic capacity [27]. An increase in the relative proportion of* Firmicutes* is linked to obesity as* Firmicutes* ferments plant polysaccharides to produce short-chain fatty acids (SCFA), which provides additional energy for the host [28]. In phyla, IFNT supplementation decreases the percentage of* Firmicutes*, while increasing the* Bacteroidetes* in the jejunum and ileum. However, IFNT supplementation increases the percentage of* Firmicutes*, while decreasing the* Bacteroidetes* in the colon and feces. Thus, IFNT supplementation may regulate body weight and metabolism through effects on the intestinal microbiota. At the genus level, IFNT supplementation decreases the* Lactobacillus* in the jejunum and ileum but increases the percentage of* Lactobacillus* and* Bacteroides* in the colon and feces*. Lactobacillus* has critical roles in the intestine to combat gastrointestinal bacterial pathogens and rotaviruses through competitive metabolic interactions and the production of antimicrobial molecules [29].* Bacteroides* are known for their capacity to metabolize a wide variety of oligosaccharides from the intestinal luminal, such as xylan, starch, and host-derived glycans [30]. Thus, results of the present study suggest that IFNT supplementation affects those functions of the intestinal microbiome in mice.

IFNT supplementation inhibits intestinal expression of IL-17, which suggests that IFNT reduces intestinal inflammation. IL-17 is produced by inducible Th17 (iTh17) cells and natural Th17 (nTh17) cells and regarded as an intestinal proinflammatory cytokine [31]. IL-17 can activate nuclear factor *κ*B (NF-*κ*B) transcription factors, extracellular signal-regulated protein kinase (ERK1 and ERK2), c-Jun N-terminal kinases (JNK-1 and JNK-2), and mitogen-activated protein kinases (p38 MAPKs) pathways, leading to upregulation of expression of inflammatory cytokines, such as IL-6 and IL-1 [32]. Recent investigations have revealed that mammalian target of rapamycin (mTOR) is a critical signaling pathway for Th17 responses and IL-17 expression [33–37]. The mTOR signaling regulates IL-17 expression through hypoxia-inducible factor 1 *α* (HIF-1*α*) and ribosomal protein S6 kinase (S6K: S6K1 and S6K2) [37–40]. mTOR signaling activates HIF-1*α*, which promotes IL-17 expression by activating ROR*γ*t (a key transcriptional regulator of Th17 cells) and mediating degradation of Foxp3 (a key transcriptional regulator of Treg cells) [40]. S6K1 promotes the expression of early growth response protein 2 (EGR2), which then inhibits growth factor independent 1 transcription repressor (GFI1), which can negatively regulate expression of IL-17 without affecting* Rorc* expression [37, 38]. S6K2 (the nuclear-localized counterpart of S6K1) binds to ROR*γ*t to promote nuclear translocation of ROR*γ*t, which can complex with HIF-*α* and p300 in the nucleus to promote expression of IL-17 [37–39]. Thus, the underlying mechanism by which IFNT supplementation reduces intestinal IL-17 expression is of interest. The effect of IFNT supplementation to decrease expression of IL-17 in the intestine indicates a potential therapeutic application of IFNT to mitigate intestinal inflammatory diseases associated with expression of IL-17.

In conclusion, IFNT supplementation affects the diversity and composition of the intestinal microbiota and decreases expression of IL-17 in mice. The findings from this study are significant in understanding the physiological and immunological functions of IFNT in treatment of inflammatory diseases.

## Figures and Tables

**Table 1:** Comparison of phylotype coverage and diversity estimation of the 16S rDNA gene libraries at 97% similarity from the pyrosequencing analysis.

Group	Number of readings	Number of OTU	Coverage	Richness estimator		Diversity index	
				Ace (95% CI)	Chao (95% CI)	Shannon (95% CI)	Simpson (95% CI)
Jejunum							
Control	12323	51	99.85%	78 (62–120)	70 (57–108)	1.06 (1.04–1.09)	0.52 (0.51–0.53)
IFNT	15653	64	99.95%	70 (66–83)	67 (65–78)	1.32 (1.30–1.35)	0.44 (0.43–0.45)

Ileum							
Control	12442	37	99.89%	52 (42–84)	57 (42–110)	0.73 (0.70–0.75)	0.70 (0.69–0.71)
IFNT	12264	66	99.79%	124 (99–167)	174 (101–400)	1.06 (1.03–1.08)	0.56 (0.55–0.56)

Colon							
Control	10327	298	99.58%	323 (311–343)	322 (309–349)	4.32 (4.29–4.35)	0.032 (0.030–0.034)
IFNT	11276	288	99.68%	306 (297–324)	313 (299–345)	4.32 (4.29–4.34)	0.027 (0.026–0.028)

Feces							
Control	12613	314	99.69%	336 (326–356)	339 (325–369)	4.39 (4.36–4.41)	0.026 (0.025–0.027)
IFNT	12493	312	99.58%	345 (331–371)	363 (337–414)	4.16 (4.13–4.19)	0.046 (0.044–0.049)

## Abbreviations

EGR2: Early growth response protein 2
ERK: Extracellular signal-regulated protein kinase
ETEC: Enterotoxigenic* Escherichia coli*
FoxP3: Forkhead box P3
GFI1: Growth factor independent 1 transcription repressor
HIF-1*α*: Hypoxia-inducible factor 1*α*
mTOR: Mammalian target of rapamycin
ROR*γ*t: Retinoic acid receptor-related orphan receptor gamma t
S6K: Ribosomal protein S6 kinase.
